# A Guiding Principle for Quantum State Discrimination in the Real-Spectrum Phase of *P*-Pseudo-Hermitian Systems

**DOI:** 10.3390/e27080836

**Published:** 2025-08-06

**Authors:** Qinliang Dong, Xueer Gao, Zhihang Liu, Hui Li, Jingwei Wen, Chao Zheng

**Affiliations:** 1 School of Energy Storage Science and Engineering, North China University of Technology, Beijing 100144, China; 2 Integrated Circuit College, Beijing Polytechnic University, Beijing 100176, China; 3 China Mobile (Suzhou) Software Technology Company Limited, Suzhou 215163, China; 4 Beijing Laboratory of New Energy Storage Technology, Beijing 100144, China

**Keywords:** quantum state discrimination, non-Hermitian, *P*-pseudo-Hermitian, PT-symmetric

## Abstract

Quantum state discrimination (QSD) is a fundamental task in quantum information processing, improving the computation efficiency and communication security. Non-Hermitian (NH) PT-symmetric systems were found to be able to discriminate two quantum states better than the Hermitian strategy. In this work, we propose a QSD approach based on *P*-pseudo-Hermitian systems with real spectra. We theoretically prove the feasibility of realizing QSD in the real-spectrum phase of a *P*-pseudo-Hermitian system, i.e., two arbitrary non-orthogonal quantum states can be discriminated by a suitable *P*-pseudo-Hermitian Hamiltonian. In detail, we decide the minimal angular separation between two non-orthogonal quantum states for a fixed *P*-pseudo-Hermitian Hamiltonian, and we find the orthogonal evolution time is able to approach zero under suitable conditions, while both the trace distance and the quantum relative entropy are employed to judge their orthogonality. We give a criterion to choose the parameters of a *P*-pseudo-Hermitian Hamiltonian that evolves the two initial orthogonal states faster than a fixed arbitrary PT-symmetric one with an identical energy difference. Our work expands the NH family for QSD, and can be used to explore real quantum systems in the future.

## 1. Introduction

Quantum state discrimination (QSD) [1] is a fundamental problem that underpins a wide range of applications in quantum information processing [2,3,4,5]. Alice and Bob agree on a set of quantum states, with the prior probability of each state known in advance. Alice then encodes information into these states and sends them to Bob, who needs to distinguish among the states in order to decode the transmitted information [2]. According to the fundamental principles of quantum mechanics, a single quantum measurement can extract only limited information, making it impossible to unambiguously discriminate between arbitrary non-orthogonal states with unit success probability [1,6,7]. This fundamental limitation has motivated researchers to explore various optimal QSD strategies, aiming to maximize the success probability of correct identification by designing an appropriate measurement strategy under given prior probabilities and operational constraints. In the context of Hermitian quantum mechanics, there are two basic QSD strategies: minimum error discrimination (MED) [8,9,10] and unambiguous state discrimination (USD) [11,12,13,14,15,16,17]. MED is never conclusive and aims to minimize the average probability of drawing the wrong conclusion, whereas USD can ascertain the state with a non-zero probability of success. At present, the optimal solutions for MED and USD strategies in Hermitian systems are limited to specific sets of non-orthogonal quantum states [18]. A universal optimal solution is still required for the discrimination of arbitrary non-orthogonal quantum states.

With the development of non-Hermitian (NH) quantum mechanics, it has attracted widespread research interesting from various areas, such as NH quantum information processing [19,20,21,22,23,24,25,26,27,28,29,30,31,32,33,34,35,36,37,38,39,40,41,42,43,44], open and dissipative quantum systems [45,46,47,48,49,50,51], etc. In fact, NH systems have intrinsic connections to QSD, which is a non-trivial problem only for non-orthogonal quantum states, say $|\psi_{1}\rangle$ and $|\psi_{2}\rangle$. QSD of the two states can be achieved if the two non-orthogonal states $|\psi_{1}\rangle$ and $|\psi_{2}\rangle$ are evolved into two orthogonal final states. It cannot be achieved by Hermitian systems directly, because a Hermitian Hamiltonian leads to unitary evolution, keeping the inner product of two non-orthogonal quantum states invariant. However, NH Hamiltonians can realize non-unitary evolutions, which can evolve the two initial states into orthogonal ones. This fact gives non-Hermitian systems an inbuilt advantage over the Hermitian systems to solve the QSD directly. Analogous to the USD strategies in Hermitian systems, we develop an NH discrimination strategy of which the key point is the orthogonalization of two non-orthogonal initial states under an NH Hamiltonian *H*. Our NH discrimination strategy is able to deterministically discriminate the two initial states in a single measurement by orthogonalizing them.

Since PT-symmetric NH systems were discovered [52,53,54], they have attracted widespread attention in recent years. In a PT-symmetric system, the Hamiltonian is no longer required to be Hermitian but is instead required to commute with the combined PT operator, i.e., $[H_{PT},PT]=0$, where *P* is the parity reflection operator and *T* is the time-reversal operator. Although a PT-symmetric Hamiltonian is NH, its eigenvalues remain real within the PT-unbroken phase. In [55], the discrimination of two non-orthogonal quantum states was realized using a PT-symmetric Hamiltonian. The discrimination of two and three non-orthogonal quantum states was experimentally realized in a PT-symmetric system using a linear optical setup [56]. The paper [57] pointed out that in Hermitian systems, the time required for an initial state to evolve into a final state is finite and non-zero. This is a quantum analogue of the classical brachistochrone problem [58,59]. In PT-symmetric systems, when the energy difference (i.e., the difference between the maximum and minimum eigenvalues of the Hamiltonian) is fixed, the required evolution time can approach zero as the Hamiltonian elements become sufficiently large [55,56], a phenomenon that has also been demonstrated experimentally [60]. The paper [61] theoretically extended the quantum brachistochrone to the PT symmetry-broken regime.

Coherence time is a highly limited resource in current and near-term quantum computers of the Noisy Intermediate-Scale Quantum (NISQ) era [62]. Therefore, faster evolution is crucial for state preparation and QSD. Although PT-symmetric quantum systems theoretically allow for arbitrarily fast evolution, this requires infinitely large parameters. Explicitly, consider the two-level PT-symmetric Hamiltonian $H_{PT}=\left(\begin{array}{cc} re^{i\theta } & k\\ k & re^{-i\theta } \end{array}\right)$ with a fixed energy difference ω, the time needed for evolving $\left(\begin{array}{c} 1\\ 0 \end{array}\right)$ to $\left(\begin{array}{c} 0\\ 1 \end{array}\right)$ under $H_{PT}$ is t=(2α+π)ℏ/ω with sinα=rsinθ/s. In the limit α→−π/2, we have t→0. The fixed energy difference ω can be expressed as $\omega^{2}=4s^{2}\cos^{2}\alpha$ and as ω is fixed, α→−π/2 requires s→∞ [57]. In the experimental study of fast evolution in PT-symmetric quantum systems, experiments are implemented for several α values in the range of [−31π/64,0] [60]. It is pointed out that in the QSD problem, the limit α→−π/2 corresponds to applying a magnetic field in a complex direction with the imaginary component of this magnetic field rsinθ→∞, and the experimental realization remains challenging [55]. Given this constraint, it is worth theoretically investigating alternative approaches that can achieve faster evolution for QSD than PT-symmetric systems under the same energy constraint.

Similar to PT-symmetric systems, the eigenvalues of a pseudo-Hermitian system can also be real. Subsequent studies have shown that pseudo-Hermiticity is, in fact, a necessary and sufficient condition for a Hamiltonian to have real eigenvalues [63,64,65]. A η-pseudo-Hermitian Hamiltonian *H* satisfies the relation $H^{†}=\eta H\eta^{-1}$ [65,66,67,68,69]; η is usually required to be a linear Hermitian operator. In [70], a pseudo-Hermitian system is used to discriminate a specific pair of slightly different entangled states.

In this work, we focus on two-level quantum systems. By specifying the operator η with the parity operator *P*, a *P*-pseudo-Hermitian Hamiltonian is obtained. Our work extends the QSD method to the real-spectrum phase of *P*-pseudo-Hermitian systems. We prove that two arbitrary non-orthogonal states can be evolved into orthogonal states, and we employ both the trace distance and the quantum relative entropy to judge their orthogonality, under the time evolution operator governed by a suitable *P*-pseudo-Hermitian Hamiltonian. A real binary measurement can be used to discriminate the states with certainty. Under appropriate conditions, the evolution time can approach zero. For a fixed *P*-pseudo-Hermitian Hamiltonian, we decide the minimal angular separation between two initial states that can be evolved into orthogonal ones. We give a criterion that how to choose the parameters of a *P*-pseudo-Hermitian Hamiltonian that orthogonally evolves the two states faster than an arbitrarily fixed PT-symmetric Hamiltonian with an identical energy difference. Under the condition of equal energy difference, we further obtain a simplified criterion when the parameters of the *P*-pseudo-Hermitian and PT-symmetric Hamiltonians satisfy specific conditions.

## 2. Two-State Discrimination in *P*-Pseudo-Hermitian and PT-Symmetric Systems

We consider a two-dimensional subspace spanned by the quantum states $|\psi_{1}\rangle$ and $|\psi_{2}\rangle$. Assume the angular separation between these two states on the Bloch sphere is 2ε. Without loss of generality, we can reparameterize the Bloch sphere such that both states lie along the same meridian. Specifically, let $|\psi_{1}\rangle$ be at the spherical coordinates $\left(\varphi ,\;\phi \right)$, and $|\psi_{2}\rangle$ at $\left(\varphi +2\varepsilon ,\;\phi \right)$. In this case, the two states can be expressed as

(1) $$|\psi_{1}\rangle =\left(\begin{array}{c} \cos\frac{\varphi }{2}\\ e^{i\phi }\sin\frac{\varphi }{2} \end{array}\right)\mathrm{and}\;|\psi_{2}\rangle =\left(\begin{array}{c} \cos\frac{\varphi +2\varepsilon }{2}\\ e^{i\phi }\sin\frac{\varphi +2\varepsilon }{2} \end{array}\right).$$

To simplify, we choose $\phi =-\frac{\pi }{2}$ and $\varphi =\frac{\pi }{2}-\varepsilon$. Substituting these values, the two states become

(2) $$|\psi_{1}\rangle =\left(\begin{array}{c} \cos\frac{\pi -2\varepsilon }{4}\\ -i\sin\frac{\pi -2\varepsilon }{4} \end{array}\right)\mathrm{and}\;|\psi_{2}\rangle =\left(\begin{array}{c} \cos\frac{\pi +2\varepsilon }{4}\\ -i\sin\frac{\pi +2\varepsilon }{4} \end{array}\right),$$

where $\varepsilon \in (0,\frac{\pi }{2})$.

We will investigate how to discriminate the two quantum states above using two-level *P*-pseudo-Hermitian systems and PT-symmetric systems with the same energy difference, respectively, after we introduce the two non-Hermitian quantum systems below.

### 2.1. QSD Using a P-Pseudo-Hermitian Hamiltonian

We take two-level quantum systems; for example, a *P*-pseudo-Hermitian Hamiltonian, satisfying $PH_{PPH}^{†}P=H_{PPH}$, can be expressed as

(3) $$H_{PPH}=\left(\begin{array}{cc} re^{i\theta } & s\\ u & re^{-i\theta } \end{array}\right)=r\cos\theta 1+\vec{\sigma }\cdot \left(\frac{s+u}{2},i\frac{s-u}{2},ir\sin\theta \right),$$

where the capital *P* is short for parity and here we set $P=\sigma_{1}$; the parameters *s*, *u*, *r* and θ are real numbers, 1 is the identity matrix, and $\vec{\sigma }$ represents the Pauli matrices

(4) $$\sigma_{1}=\left(\begin{array}{cc} 0 & 1\\ 1 & 0 \end{array}\right),\;\sigma_{2}=\left(\begin{array}{cc} 0 & -i\\ i & 0 \end{array}\right)\mathrm{and}\;\sigma_{3}=\left(\begin{array}{cc} 1 & 0\\ 0 & -1 \end{array}\right).$$

The eigenvalues of $H_{PPH}$ are given by

(5) $$E_{PPH\pm }=r\cos\theta \pm \sqrt{su-r^{2}\sin^{2}\theta },$$

The condition $su>r^{2}\sin^{2}\theta$ ensures that the eigenvalues are real. This condition can alternatively be expressed in terms of sinα as

(6) $$\sin\alpha =\frac{r\sin\theta }{\sqrt{su}}\in \left(-1,1\right).$$

The difference between the two eigenvalues is given by

(7) $$\omega =\sqrt{su-r^{2}\sin^{2}\theta }.$$

Through calculation, we obtain the time evolution operator $U_{PPH}$ governed by $H_{PPH}$ is

(8) $$U_{PPH}\left(t\right)=e^{-\frac{iH_{PPH}t}{\hbar }}=\frac{e^{-irt\cos\theta }}{\cos\alpha }\left(\begin{array}{cc} \cos(\omega t-\alpha ) & -i\sin\left(\omega t\right)\frac{\sqrt{su}}{u}\\ -i\sin\left(\omega t\right)\frac{\sqrt{su}}{s} & \cos(\omega t+\alpha ) \end{array}\right),$$

where we set ℏ=1. For the initially non-orthogonal states $|\psi_{1}\rangle$ and $|\psi_{2}\rangle$, the inner product of their time-evolved states under $U_{PPH}$ is given by (see Appendix A for details)

(9) $$\begin{array}{cc} \; & \langle \psi_{1}|U_{PPH}^{†}U_{PPH}|\psi_{2}\rangle \\ = & \frac{\sin^{2}\left(\omega t\right)\left(-\cos\varepsilon \cos\left(2\alpha \right)-\frac{s+u}{\sqrt{su}}\sin\alpha +\frac{s^{2}+u^{2}}{2su}\cos\varepsilon \right)+\frac{u-s}{2\sqrt{su}}\cos\alpha \sin\left(2\omega t\right)+\cos^{2}\alpha \cos\varepsilon }{\cos^{2}\alpha }. \end{array}$$

The inner product vanishes when

(10) $$\sin^{2}\left(\omega t\right)=\frac{a^{2}+b^{2}+ac\pm \sqrt{b^{2}\left(a^{2}+b^{2}-c^{2}\right)}}{2\left(a^{2}+b^{2}\right)},$$

where we set

(11) $$\begin{array}{cc} \; & a=\cos\varepsilon \cos\left(2\alpha \right)+\frac{s+u}{\sqrt{su}}\sin\alpha -\frac{s^{2}+u^{2}}{2su}\cos\varepsilon ,\\ \; & b=\frac{u-s}{\sqrt{su}}\cos\alpha ,\\ \; & c=\frac{{\left(s+u\right)}^{2}}{2su}\cos\varepsilon -\frac{s+u}{\sqrt{su}}\sin\alpha . \end{array}$$

From Equation (10), the possible evolution times within one period are given by

(12) $$t_{1},\;t_{2},\;\frac{\pi }{\omega }-t_{1},\;\frac{\pi }{\omega }-t_{2},$$

where $t_{1}$ and $t_{2}$ are given by

(13) $$t_{1}=\frac{\arcsin\left(\sqrt{\frac{a^{2}+b^{2}+ac+\sqrt{b^{2}\left(a^{2}+b^{2}-c^{2}\right)}}{2\left(a^{2}+b^{2}\right)}}\right)}{\omega },\;t_{2}=\frac{\arcsin\left(\sqrt{\frac{a^{2}+b^{2}+ac-\sqrt{b^{2}\left(a^{2}+b^{2}-c^{2}\right)}}{2\left(a^{2}+b^{2}\right)}}\right)}{\omega }.$$

According to the discussion in Appendix A on the existence of non-trivial solutions for *t*, the time-evolved states $U_{PPH}|\psi_{1}\rangle$ and $U_{PPH}|\psi_{2}\rangle$ become orthogonal twice within one period. The corresponding evolution times are one of $t_{1}$ and $\frac{\pi }{\omega }-t_{1}$, and one of $t_{2}$ and $\frac{\pi }{\omega }-t_{2}$. When the values of *s* and *u* are interchanged while keeping other parameters unchanged, the evolution times will correspond to another set of solutions. The condition for $t_{1}$ and $t_{2}$ to be non-trivial solutions is $a^{2}+b^{2}-c^{2}\geq 0$, from which we obtain

(14) $$\cos\varepsilon \leq \frac{\frac{s+u}{\sqrt{su}}\sin\alpha +\sqrt{\frac{s^{2}+u^{2}}{2su}\left[\frac{{\left(s+u\right)}^{2}}{su}-4\cos^{2}\alpha \right]}}{\frac{{\left(s+u\right)}^{2}}{su}-2\cos^{2}\alpha },$$

at the critical value

(15) $$\cos\varepsilon =\frac{\frac{s+u}{\sqrt{su}}\sin\alpha +\sqrt{\frac{s^{2}+u^{2}}{2su}\left[\frac{{\left(s+u\right)}^{2}}{su}-4\cos^{2}\alpha \right]}}{\frac{{\left(s+u\right)}^{2}}{su}-2\cos^{2}\alpha },$$

we have $t_{1}=t_{2}$. For arbitrary initially non-orthogonal states $|\psi_{1}\rangle$ and $|\psi_{2}\rangle$, as long as Equation (14) is satisfied, they can be evolved into orthogonal ones in a *P*-pseudo-Hermitian system.

We next study the conditions under which the evolution time approaches zero. We fix the energy difference ω and consider the limit where either s→∞ or u→∞. In this limit, r→∞, $\cos\alpha =\frac{\omega }{\sqrt{su}}\to 0$. Furthermore, it can be obtained that a→∞, *b* tends to a constant value, c→∞, and the sum satisfies $a+c=2\cos^{2}\alpha \cos\varepsilon \to 0$. From Equation (13), we obtain that the evolution times $t_{1}\to 0$ and $t_{2}\to 0$.

### 2.2. QSD Using a PT-Symmetric Hamiltonian

The Hamiltonian of the PT-symmetric two-level system is given by

(16) $$H_{PT}=\left(\begin{array}{cc} re^{i\theta } & k\\ k & re^{-i\theta } \end{array}\right)=r\cos\theta 1+\vec{\sigma }\cdot \left(k,0,ir\sin\theta \right),$$

where *P* and *T* are the parity and time-reversal operations; the parameters *k*, *r* and θ are real numbers. The eigenvalues of $H_{PT}$ are given by

(17) $$E_{\pm }=r\cos\theta \pm \sqrt{k^{2}-r^{2}\sin^{2}\theta },$$

the condition $k^{2}>r^{2}\sin^{2}\theta$ ensures that the eigenvalues are real. This condition can alternatively be expressed in terms of sinβ as

(18) $$\sin\beta =\frac{r\sin\theta }{k}\in \left(-1,1\right).$$

The difference between the two eigenvalues is given by

(19) $$\Delta =\sqrt{k^{2}-r^{2}\sin^{2}\theta }.$$

The time evolution operator governed by $H_{PT}$ is

(20) $$U_{PT}\left(t\right)=e^{-\frac{iH_{PT}t}{\hbar }}=\frac{e^{-irt\cos\theta }}{\cos\beta }\left(\begin{array}{cc} \cos(\Delta t-\beta ) & -i\sin(\Delta t)\\ -i\sin(\Delta t) & \cos(\Delta t+\beta ) \end{array}\right),$$

where we set ℏ=1. For the initially non-orthogonal states $|\psi_{1}\rangle$ and $|\psi_{2}\rangle$, the inner product of their time-evolved states under $U_{PT}$ is given by

(21) $$\langle \psi_{1}|U_{PT}^{†}U_{PT}|\psi_{2}\rangle =\frac{2\sin^{2}\left(\Delta t\right)\left(\sin^{2}\beta \cos\varepsilon -\sin\beta \right)+\cos\varepsilon \cos^{2}\beta }{\cos^{2}\beta },$$

the inner product vanishes when

(22) $$\sin^{2}\left(\Delta t\right)=\frac{\cos^{2}\beta \cos\varepsilon }{2\sin\beta -2\sin^{2}\beta \cos\varepsilon },$$

we can obtain the evolution times within one period

(23) $$t_{3}=\frac{\arcsin\left(\sqrt{\frac{\cos^{2}\beta \cos\varepsilon }{2\sin\beta -2\sin^{2}\beta \cos\varepsilon }}\right)}{\Delta },\;\frac{\pi }{\Delta }-t_{3}.$$

The non-trivial solution $t_{3}$ must satisfy the following inequality:

(24) $$\cos\varepsilon \leq \frac{2\sin\beta }{1+\sin^{2}\beta },$$

at the critical value

(25) $$\cos\varepsilon =\frac{2\sin\beta }{1+\sin^{2}\beta },$$

we have $t_{3}=\frac{\pi }{2\Delta }$. For arbitrary initially non-orthogonal states $|\psi_{1}\rangle$ and $|\psi_{2}\rangle$, as long as Equation (24) is satisfied, they can be evolved into orthogonal states in a PT-symmetric system. Under the condition of a fixed energy difference Δ, when the parameters k→∞ and r→∞, we have cosβ→0. According to Equation (23), we have $t_{3}\to 0$. This indicates that the quantum brachistochrone can be realized in the real-spectrum regime of the PT-symmetric system.

In fact, both the PT-symmetric and *P*-pseudo-Hermitian systems are able to achieve QSD, attributed to the novel properties of NH physics. In different cases, either PT-symmetric or *P*-pseudo-Hermitian systems would perform better to discriminate two similar initial quantum states, as long as the parameters of the NH systems are designed properly. In the next section, under the condition of equal energy difference, we provide a criterion for selecting the *P*-pseudo-Hermitian Hamiltonian that can evolve the initial states $|\psi_{1}\rangle$ and $|\psi_{2}\rangle$ into orthogonal ones faster than any fixed PT-symmetric Hamiltonian.

## 3. The Time Required for the Two Systems to Evolve $|\psi_{1}\rangle$ and $|\psi_{2}\rangle$ into Orthogonal Ones

In search of more efficient methods for QSD, we compare the time required for the initial states $|\psi_{1}\rangle$ and $|\psi_{2}\rangle$ to evolve into orthogonal ones in *P*-pseudo-Hermitian systems and PT-symmetric systems. We set the two systems to have an identical energy difference, i.e., ω=Δ. When the condition

(26) $$\min\left\{t_{1},t_{2}\right\}<t_{3},$$

is satisfied, it indicates that the states $U_{PPH}|\psi_{1}\rangle$ and $U_{PPH}|\psi_{2}\rangle$ become orthogonal faster than $U_{PT}|\psi_{1}\rangle$ and $U_{PT}|\psi_{2}\rangle$. To quantify the distinguishability between time-evolved states, we employ two measures: the trace distance and the quantum relative entropy.

The trace distance is defined as

(27) $$D\left(\rho ,\sigma \right)=\frac{1}{2}\mathrm{Tr}\left|\rho -\sigma \right|,$$

where $\left|A\right|=\sqrt{A^{†}A}$, ρ and σ represent the density matrices of the time-evolved states $U_{PPH}|\psi_{1}\rangle$ and $U_{PPH}|\psi_{2}\rangle$, or $U_{PT}|\psi_{1}\rangle$ and $U_{PT}|\psi_{2}\rangle$. In this study, we apply normalization to the density matrices under non-unitary evolution. Here, D∈[0,1], where D=1 indicates that the two time-evolved states are orthogonal.

For two density operators ρ and σ defined on the same Hilbert space, the quantum relative entropy is defined as

(28) S(ρ∥σ)=Tr[ρ(logρ−logσ)].

If suppρ⊆suppσ, then the quantum relative entropy S(ρ∥σ) is finite; otherwise, S(ρ∥σ) is defined to be +∞. We apply normalization and regularization to the density matrices under non-unitary evolution. During the time evolution of the initial states $|\psi_{1}\rangle$ and $|\psi_{2}\rangle$, a peak in the quantum relative entropy *S* between the two time-evolved states indicates that they become orthogonal.

**Figure 1:**
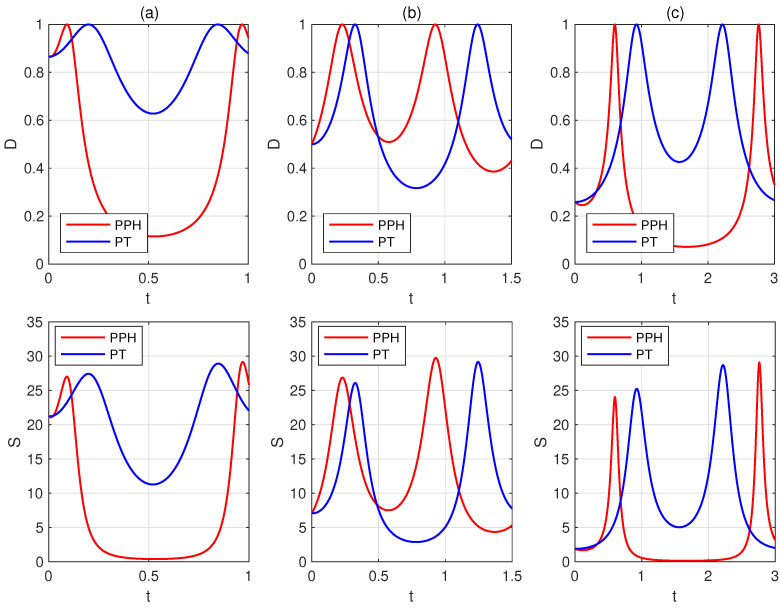
The trace distance *D* and the quantum relative entropy *S* between the states $U_{PPH}|\psi_{1}\rangle$ and $U_{PPH}|\psi_{2}\rangle$, as well as between $U_{PT}|\psi_{1}\rangle$ and $U_{PT}|\psi_{2}\rangle$, under the condition ω=Δ. The plots correspond to the following parameter sets: (**a**) s=8, u=9, ω=3, k=4, ε=π/3; (**b**) s=6, u=3, ω=2, k=5, ε=π/6; (**c**) s=7, u=8, ω=1, k=3, ε=π/12. The red (blue) lines represent the dynamical variation of the trace distance *D* or quantum relative entropy *S* between the states $U_{PPH}|\psi_{1}\rangle$ and $U_{PPH}|\psi_{2}\rangle$ ($U_{PT}|\psi_{1}\rangle$ and $U_{PT}|\psi_{2}\rangle$). The results show that the states $U_{PPH}|\psi_{1}\rangle$ and $U_{PPH}|\psi_{2}\rangle$ become orthogonal faster than $U_{PT}|\psi_{1}\rangle$ and $U_{PT}|\psi_{2}\rangle$.

Next, we selected several sets of parameters based on the proposed criterion and performed numerical simulations using the trace distance *D* and quantum relative entropy *S*. In Figure 1, the red (blue) lines represent the dynamical variation of the trace distance *D* or the quantum relative entropy *S* between the states $U_{PPH}|\psi_{1}\rangle$ and $U_{PPH}|\psi_{2}\rangle$ ($U_{PT}|\psi_{1}\rangle$ and $U_{PT}|\psi_{2}\rangle$), plotted as a function of *t*. The results show that the states $U_{PPH}|\psi_{1}\rangle$ and $U_{PPH}|\psi_{2}\rangle$ become orthogonal faster than $U_{PT}|\psi_{1}\rangle$ and $U_{PT}|\psi_{2}\rangle$. The time points at which the time-evolved states become orthogonal, obtained through numerical simulations using the trace distance *D* and quantum relative entropy *S*, are consistent.

**Figure 2:**
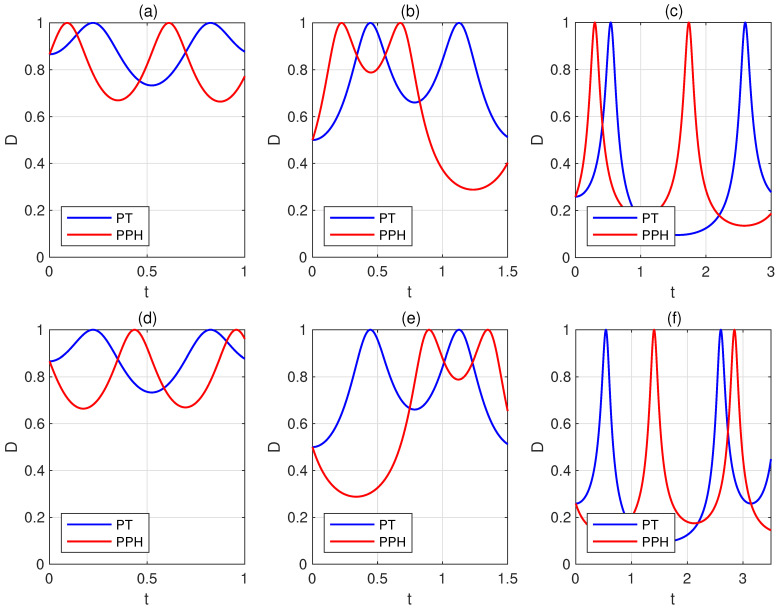
The trace distance *D* between the states $U_{PPH}|\psi_{1}\rangle$ and $U_{PPH}|\psi_{2}\rangle$, as well as between $U_{PT}|\psi_{1}\rangle$ and $U_{PT}|\psi_{2}\rangle$ are plotted as a function of *t*. We fix $\sqrt{su}=k$ and ω=Δ. The parameter sets for the top row subplots are as follows: (**a**) s=7, u=2, ω=3, $k=\sqrt{14}$, ε=π/3; (**b**) s=6, u=2, ω=2, $k=2\sqrt{3}$, ε=π/6; (**c**) s=8, u=5, ω=1, $k=2\sqrt{10}$, ε=π/12. The parameter settings for the corresponding subplots (**d**–**f**) are obtained by exchanging the values of *s* and *u* in subplots (**a**–**c**). The red (blue) lines represent the dynamical variation of the trace distance *D* between the states $U_{PPH}|\psi_{1}\rangle$ and $U_{PPH}|\psi_{2}\rangle$ ($U_{PT}|\psi_{1}\rangle$ and $U_{PT}|\psi_{2}\rangle$). The results indicate that when s>u, $U_{PPH}|\psi_{1}\rangle$ and $U_{PPH}|\psi_{2}\rangle$ become orthogonal faster than $U_{PT}|\psi_{1}\rangle$ and $U_{PT}|\psi_{2}\rangle$. Conversely, when s<u, $U_{PT}|\psi_{1}\rangle$ and $U_{PT}|\psi_{2}\rangle$ become orthogonal faster than $U_{PPH}|\psi_{1}\rangle$ and $U_{PPH}|\psi_{2}\rangle$.

**Figure 3:**
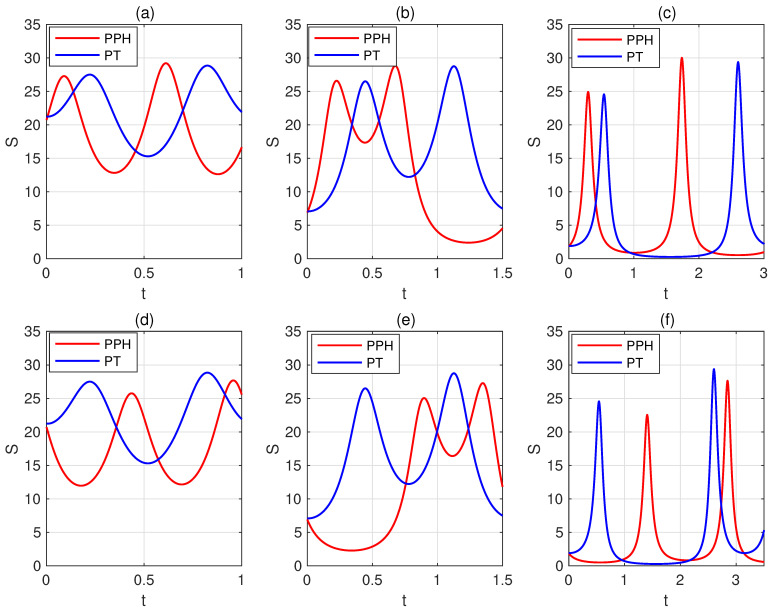
The quantum relative entropy *S* between the states $U_{PPH}|\psi_{1}\rangle$ and $U_{PPH}|\psi_{2}\rangle$, as well as between $U_{PT}|\psi_{1}\rangle$ and $U_{PT}|\psi_{2}\rangle$ are plotted as a function of *t*. We fix $\sqrt{su}=k$ and ω=Δ. The parameter sets for the top row subplots are as follows: (**a**) s=7, u=2, ω=3, $k=\sqrt{14}$, ε=π/3; (**b**) s=6, u=2, ω=2, $k=2\sqrt{3}$, ε=π/6; (**c**) s=8, u=5, ω=1, $k=2\sqrt{10}$, ε=π/12. The parameter settings for the corresponding subplots (**d**–**f**) are obtained by exchanging the values of *s* and *u* in subplots (**a**–**c**). The red (blue) lines represent the dynamical variation of the quantum relative entropy *S* between the states $U_{PPH}|\psi_{1}\rangle$ and $U_{PPH}|\psi_{2}\rangle$ ($U_{PT}|\psi_{1}\rangle$ and $U_{PT}|\psi_{2}\rangle$). The results indicate that when s>u, $U_{PPH}|\psi_{1}\rangle$ and $U_{PPH}|\psi_{2}\rangle$ become orthogonal faster than $U_{PT}|\psi_{1}\rangle$ and $U_{PT}|\psi_{2}\rangle$. Conversely, when s<u, $U_{PT}|\psi_{1}\rangle$ and $U_{PT}|\psi_{2}\rangle$ become orthogonal faster than $U_{PPH}|\psi_{1}\rangle$ and $U_{PPH}|\psi_{2}\rangle$.

Next, we give a simplified criterion by fixing $\sqrt{su}=k$ and ω=Δ. We use the same parameter settings for the corresponding subplots in Figure 2 and Figure 3; the red (blue) lines represent the dynamical variation of the trace distance *D* or quantum relative entropy *S* between the states $U_{PPH}|\psi_{1}\rangle$ and $U_{PPH}|\psi_{2}\rangle$ ($U_{PT}|\psi_{1}\rangle$ and $U_{PT}|\psi_{2}\rangle$), plotted as a function of *t*. The parameter settings for the corresponding subplots (d–f) are obtained by exchanging the values of *s* and *u* in subplots (a–c). We find that when s>u, $U_{PPH}|\psi_{1}\rangle$ and $U_{PPH}|\psi_{2}\rangle$ become orthogonal faster than $U_{PT}|\psi_{1}\rangle$ and $U_{PT}|\psi_{2}\rangle$. Conversely, when s<u, $U_{PT}|\psi_{1}\rangle$ and $U_{PT}|\psi_{2}\rangle$ become orthogonal faster than $U_{PPH}|\psi_{1}\rangle$ and $U_{PPH}|\psi_{2}\rangle$. We provide the proof in Appendix B, acknowledging that this is not a complete proof, as the final result depends on δ being sufficiently small. This indicates that our findings hold under suitable conditions. A deeper explanation of this phenomenon remains a problem worthy of further investigation.

## 4. Discussion and Conclusions

In [56], the experimental discrimination of two and three non-orthogonal quantum states has been demonstrated in a PT-symmetric system using a linear optical setup. Our proposed QSD method based on the *P*-pseudo-Hermitian system is based on the quantum circuit model and can be implemented on a general quantum computer or quantum simulator. Therefore, our proposed method can be implemented in principle in any current and even future real experimental platforms based on a quantum circuit model, e.g., quantum optics, superconductors, ultra-cold atoms, trapped ions, NMR, etc.

Discriminating two non-orthogonal states plays a crucial role in quantum information theory. In this work, we extend the method of QSD to *P*-pseudo-Hermitian unbroken phase. We theoretically prove that, for two arbitrary initial non-orthogonal states $|\psi_{1}\rangle$ and $|\psi_{2}\rangle$, they can be discriminated by an appropriate *P*-pseudo-Hermitian Hamiltonian. Furthermore, we find the evolution time is able to approach zero under suitable conditions. For a given *P*-pseudo-Hermitian Hamiltonian, we give the minimal angular separation between two non-orthogonal states $|\psi_{1}\rangle$ and $|\psi_{2}\rangle$ that can be evolved into orthogonal ones. We give a criterion for choosing the parameters of a *P*-pseudo-Hermitian Hamiltonian such that it evolves the two non-orthogonal states $|\psi_{1}\rangle$ and $|\psi_{2}\rangle$ into orthogonal ones faster than an arbitrary PT-symmetric Hamiltonian with an identical energy difference. Under the condition of equal energy difference, we further obtain a simplified criterion when the parameters of $H_{PPH}$ and $H_{PT}$ satisfy $\sqrt{su}=k$. Our research provides new insights for developing efficient and resource-saving QSD strategies and broadens the applications of NH systems in quantum information processing. An interesting direction for further research is to extend the QSD method to complex-spectrum regions.

## Figures and Tables

**Figure A1:**
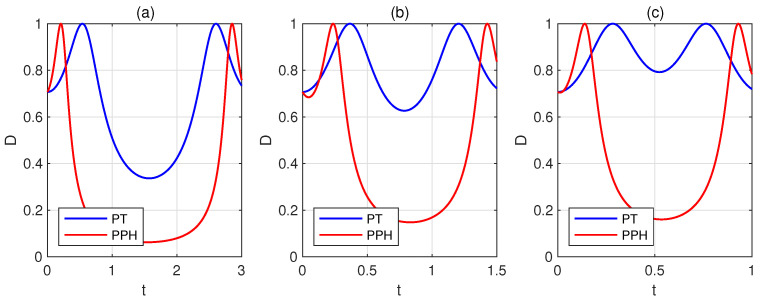
The trace distance *D* between the states $U_{PPH}|\psi_{1}\rangle$ and $U_{PPH}|\psi_{2}\rangle$, as well as between $U_{PT}|\psi_{1}\rangle$ and $U_{PT}|\psi_{2}\rangle$ are plotted as a function of *t*. We set ω=Δ and ε=π/4. The plots correspond to the following sets of parameters: (**a**) s=5, u=4, ω=1, k=2; (**b**) s=5, u=7, ω=2, k=3; (**c**) s=8, u=9, ω=3, k=4. The red (blue) lines represent the dynamical variation of the trace distance *D* between the states $U_{PPH}|\psi_{1}\rangle$ and $U_{PPH}|\psi_{2}\rangle$ ($U_{PT}|\psi_{1}\rangle$ and $U_{PT}|\psi_{2}\rangle$). That the red line reaches D=1 earlier than the blue line implies that $U_{PPH}|\psi_{1}\rangle$ and $U_{PPH}|\psi_{2}\rangle$ reach orthogonality faster than $U_{PT}|\psi_{1}\rangle$ and $U_{PT}|\psi_{2}\rangle$.

**Figure A2:**
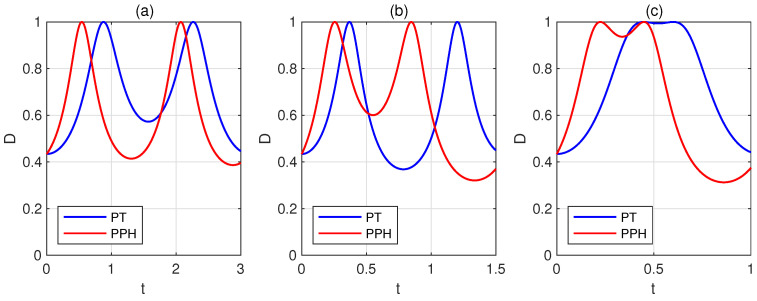
The trace distance *D* between the states $U_{PPH}|\psi_{1}\rangle$ and $U_{PPH}|\psi_{2}\rangle$, as well as between $U_{PT}|\psi_{1}\rangle$ and $U_{PT}|\psi_{2}\rangle$ are plotted as a function of *t*. We set ω=Δ and ε=π/7. The plots correspond to the following sets of parameters: (**a**) s=3, u=2, ω=1, k=2; (**b**) s=6, u=3, ω=2, k=5; (**c**) s=7, u=3, ω=3, k=4. The red (blue) lines represent the dynamical variation of the trace distance *D* between the states $U_{PPH}|\psi_{1}\rangle$ and $U_{PPH}|\psi_{2}\rangle$ ($U_{PT}|\psi_{1}\rangle$ and $U_{PT}|\psi_{2}\rangle$). That the red line reaches D=1 earlier than the blue line implies that $U_{PPH}|\psi_{1}\rangle$ and $U_{PPH}|\psi_{2}\rangle$ reach orthogonality faster than $U_{PT}|\psi_{1}\rangle$ and $U_{PT}|\psi_{2}\rangle$.

**Figure A3:**
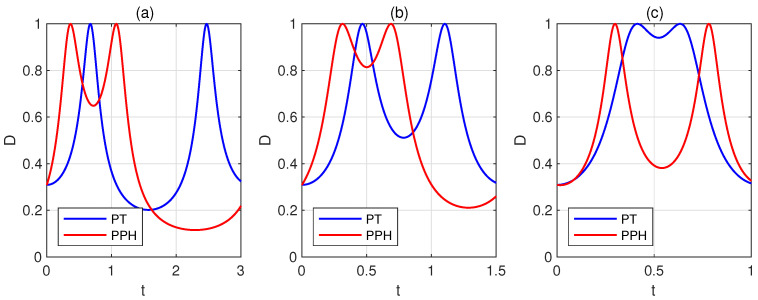
The trace distance *D* between the states $U_{PPH}|\psi_{1}\rangle$ and $U_{PPH}|\psi_{2}\rangle$, as well as between $U_{PT}|\psi_{1}\rangle$ and $U_{PT}|\psi_{2}\rangle$ are plotted as a function of *t*. We set ω=Δ and ε=π/10. The plots correspond to the following sets of parameters: (**a**) s=5, u=2, ω=1, k=4; (**b**) s=6, u=3, ω=2, k=5; (**c**) s=8.5, u=9, ω=3, k=5. The red (blue) lines represent the dynamical variation of the trace distance *D* between the states $U_{PPH}|\psi_{1}\rangle$ and $U_{PPH}|\psi_{2}\rangle$ ($U_{PT}|\psi_{1}\rangle$ and $U_{PT}|\psi_{2}\rangle$). That the red line reaches D=1 earlier than the blue line implies that $U_{PPH}|\psi_{1}\rangle$ and $U_{PPH}|\psi_{2}\rangle$ become orthogonal faster than $U_{PT}|\psi_{1}\rangle$ and $U_{PT}|\psi_{2}\rangle$.

**Figure A4:**
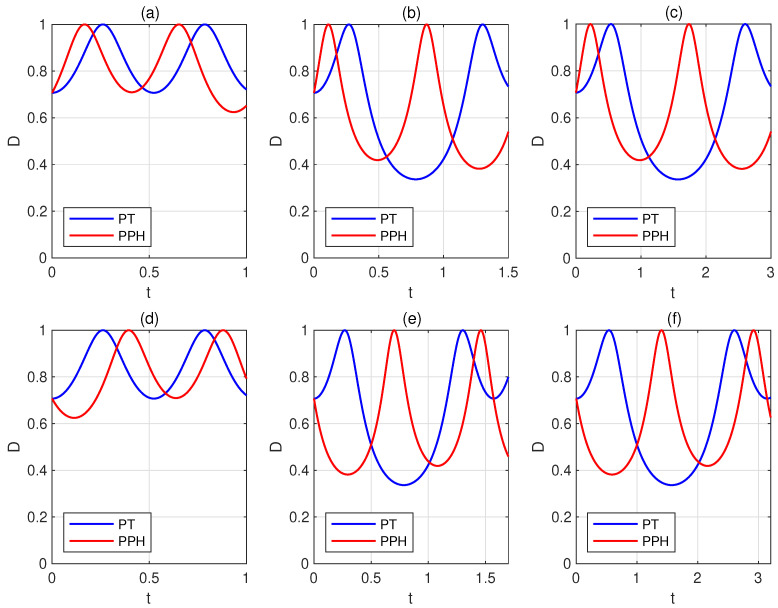
We set ε=π/4, $\sqrt{su}=k$ and ω=Δ. The trace distance *D* between the states $U_{PPH}|\psi_{1}\rangle$ and $U_{PPH}|\psi_{2}\rangle$, as well as between $U_{PT}|\psi_{1}\rangle$ and $U_{PT}|\psi_{2}\rangle$ are plotted as a function of *t*. The parameter sets for the top row subplots are as follows: (**a**) s=6, u=3, ω=3, $k=3\sqrt{2}$; (**b**) s=8, u=2, ω=2, k=4; (**c**) s=4, u=1, ω=1, k=2. The parameter settings for the corresponding subplots (**d**–**f**) are obtained by exchanging the values of *s* and *u* in subplots (**a**–**c**). The red (blue) lines represent the dynamical variation of the trace distance *D* between the states $U_{PPH}|\psi_{1}\rangle$ and $U_{PPH}|\psi_{2}\rangle$ ($U_{PT}|\psi_{1}\rangle$ and $U_{PT}|\psi_{2}\rangle$). The results indicate that when s>u, $U_{PPH}|\psi_{1}\rangle$ and $U_{PPH}|\psi_{2}\rangle$ become orthogonal faster than $U_{PT}|\psi_{1}\rangle$ and $U_{PT}|\psi_{2}\rangle$. Conversely, when s<u, $U_{PT}|\psi_{1}\rangle$ and $U_{PT}|\psi_{2}\rangle$ become orthogonal faster than $U_{PPH}|\psi_{1}\rangle$ and $U_{PPH}|\psi_{2}\rangle$.

**Figure A5:**
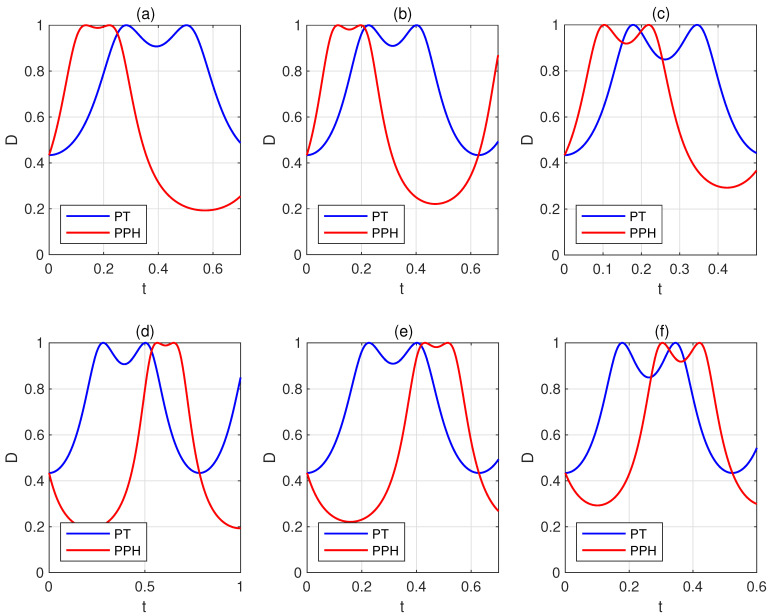
We set ε=π/7, $\sqrt{su}=k$ and ω=Δ. The trace distance *D* between the states $U_{PPH}|\psi_{1}\rangle$ and $U_{PPH}|\psi_{2}\rangle$, as well as between $U_{PT}|\psi_{1}\rangle$ and $U_{PT}|\psi_{2}\rangle$ are plotted as a function of *t*. The parameter sets for the top row subplots are as follows: (**a**) s=12, u=3, ω=4, k=6; (**b**) s=14, u=4, ω=5, $k=2\sqrt{14}$; (**c**) s=15, u=6, ω=6, $k=3\sqrt{10}$. The parameter settings for the corresponding subplots (**d**–**f**) are obtained by exchanging the values of *s* and *u* in subplots (**a**–**c**). The red (blue) lines represent the dynamical variation of the trace distance *D* between the states $U_{PPH}|\psi_{1}\rangle$ and $U_{PPH}|\psi_{2}\rangle$ ($U_{PT}|\psi_{1}\rangle$ and $U_{PT}|\psi_{2}\rangle$). The results indicate that when s>u, $U_{PPH}|\psi_{1}\rangle$ and $U_{PPH}|\psi_{2}\rangle$ become orthogonal faster than $U_{PT}|\psi_{1}\rangle$ and $U_{PT}|\psi_{2}\rangle$. Conversely, when s<u, $U_{PT}|\psi_{1}\rangle$ and $U_{PT}|\psi_{2}\rangle$ become orthogonal faster than $U_{PPH}|\psi_{1}\rangle$ and $U_{PPH}|\psi_{2}\rangle$.

**Figure A6:**
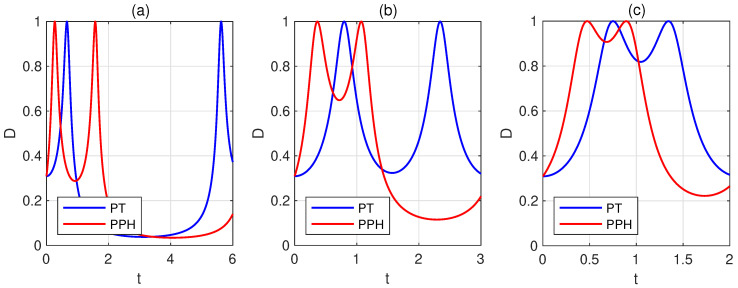

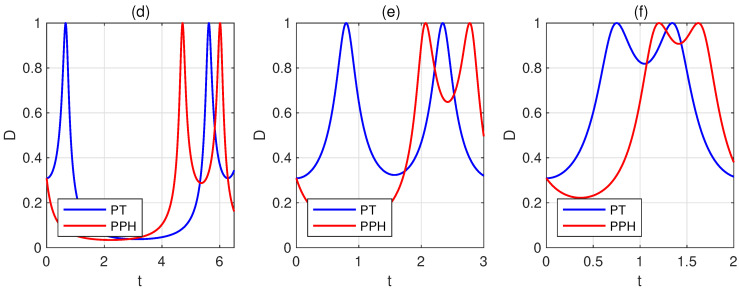
We set ε=π/10, $\sqrt{su}=k$ and ω=Δ. The trace distance *D* between the states $U_{PPH}|\psi_{1}\rangle$ and $U_{PPH}|\psi_{2}\rangle$, as well as between $U_{PT}|\psi_{1}\rangle$ and $U_{PT}|\psi_{2}\rangle$ are plotted as a function of *t*. The parameter sets for the top row subplots are as follows: (**a**) s=7, u=3, ω=0.5, $k=\sqrt{21}$; (**b**) s=5, u=2, ω=1, $k=\sqrt{10}$; (**c**) s=4, u=2, ω=1.5, $k=2\sqrt{2}$. The parameter settings for the corresponding subplots (**d**–**f**) are obtained by exchanging the values of *s* and *u* in subplots (**a**–**c**). The red (blue) lines represent the dynamical variation of the trace distance *D* between the states $U_{PPH}|\psi_{1}\rangle$ and $U_{PPH}|\psi_{2}\rangle$ ($U_{PT}|\psi_{1}\rangle$ and $U_{PT}|\psi_{2}\rangle$). The results indicate that when s>u, $U_{PPH}|\psi_{1}\rangle$ and $U_{PPH}|\psi_{2}\rangle$ become orthogonal faster than $U_{PT}|\psi_{1}\rangle$ and $U_{PT}|\psi_{2}\rangle$. Conversely, when s<u, $U_{PT}|\psi_{1}\rangle$ and $U_{PT}|\psi_{2}\rangle$ become orthogonal faster than $U_{PPH}|\psi_{1}\rangle$ and $U_{PPH}|\psi_{2}\rangle$.

## Data Availability

The original contributions presented in this study are included in the article. Further inquiries can be directed to the corresponding author(s).

## Abbreviations

The following abbreviations are used in this manuscript:

QSD	quantum state discrimination
NH	non-Hermitian
NISQ	noisy intermediate-scale quantum

## Appendix A. Details About QSD in P-Pseudo-Hermitian Systems

For the initially non-orthogonal states $|\psi_{1}\rangle$ and $|\psi_{2}\rangle$, the inner product of their time-evolved states under $U_{PPH}$ is given by

(A1) $$\begin{array}{cc} \; & \langle \psi_{1}|U_{PPH}^{†}U_{PPH}|\psi_{2}\rangle \\ = & \frac{\sin^{2}\left(\omega t\right)}{\cos^{2}\alpha }\left(-\cos\varepsilon \cos\left(2\alpha \right)-\frac{s+u}{\sqrt{su}}\sin\alpha +\frac{s^{2}+u^{2}}{2su}\cos\varepsilon \right)+\frac{u-s}{2\sqrt{su}}\frac{\cos\alpha \sin(2\omega t)}{\cos^{2}\alpha }+\frac{\cos^{2}\alpha \cos\varepsilon }{\cos^{2}\alpha }\\ = & \frac{\left(1-\cos(2\omega t)\right)\left(-\cos\varepsilon \cos\left(2\alpha \right)-\frac{s+u}{\sqrt{su}}\sin\alpha +\frac{s^{2}+u^{2}}{2su}\cos\varepsilon \right)+\frac{u-s}{\sqrt{su}}\cos\alpha \sin\left(2\omega t\right)+2\cos^{2}\alpha \cos\varepsilon }{2\cos^{2}\alpha }\\ = & \frac{\left(\cos\varepsilon \cos\left(2\alpha \right)+\frac{s+u}{\sqrt{su}}\sin\alpha -\frac{s^{2}+u^{2}}{2su}\cos\varepsilon \right)\cos\left(2\omega t\right)+\frac{u-s}{\sqrt{su}}\cos\alpha \sin\left(2\omega t\right)}{2\cos^{2}\alpha }\\ + & \frac{\left(-\frac{s+u}{\sqrt{su}}\sin\alpha +\frac{{\left(s+u\right)}^{2}}{2su}\cos\varepsilon \right)}{2\cos^{2}\alpha }=\frac{a\cos(2\omega t)+b\sin(2\omega t)+c}{2\cos^{2}\alpha }, \end{array}$$

where

(A2) $$\begin{array}{cc} \; & a=\cos\varepsilon \cos\left(2\alpha \right)+\frac{s+u}{\sqrt{su}}\sin\alpha -\frac{s^{2}+u^{2}}{2su}\cos\varepsilon ,\\ \; & b=\frac{u-s}{\sqrt{su}}\cos\alpha ,\\ \; & c=\frac{{\left(s+u\right)}^{2}}{2su}\cos\varepsilon -\frac{s+u}{\sqrt{su}}\sin\alpha . \end{array}$$

When $\langle \psi_{1}|U_{PPH}^{†}U_{PPH}|\psi_{2}\rangle =0$, we obtain

(A3) acos(2ωt)+c=−bsin(2ωt),

then

(A4) $$\left(a^{2}+b^{2}\right)\cos^{2}\left(2\omega t\right)+2ac\cos\left(2\omega t\right)+c^{2}-b^{2}=0,$$

we can obtain

(A5) $$\cos\left(2\omega t\right)=\frac{-ac\pm \sqrt{b^{2}\left(a^{2}+b^{2}-c^{2}\right)}}{a^{2}+b^{2}},$$

real solutions require $a^{2}+b^{2}-c^{2}\geq 0$, then

(A6) $$0<\cos\varepsilon \leq \frac{\frac{s+u}{\sqrt{su}}\sin\alpha +\sqrt{\frac{s^{2}+u^{2}}{2su}\left[\frac{{\left(s+u\right)}^{2}}{su}-4\cos^{2}\alpha \right]}}{\frac{{\left(s+u\right)}^{2}}{su}-2\cos^{2}\alpha },$$

where $\cos\varepsilon \in \left(0,1\right)$. Using Equation (A5), we can obtain

(A7) $$\sin^{2}\left(\omega t\right)=\frac{1-\cos(2\omega t)}{2}=\frac{a^{2}+b^{2}+ac\mp \sqrt{b^{2}\left(a^{2}+b^{2}-c^{2}\right)}}{2\left(a^{2}+b^{2}\right)},$$

the possible evolution times are

(A8) $$t_{1},\;t_{2},\;\frac{\pi }{\omega }-t_{1},\;\frac{\pi }{\omega }-t_{2},$$

where

(A9) $$\begin{array}{cc} \; & t_{1}=\frac{\arcsin\left(\sqrt{\frac{a^{2}+b^{2}+ac+\sqrt{b^{2}\left(a^{2}+b^{2}-c^{2}\right)}}{2\left(a^{2}+b^{2}\right)}}\right)}{\omega },\\ & t_{2}=\frac{\arcsin\left(\sqrt{\frac{a^{2}+b^{2}+ac-\sqrt{b^{2}\left(a^{2}+b^{2}-c^{2}\right)}}{2\left(a^{2}+b^{2}\right)}}\right)}{\omega }. \end{array}$$

According to Equation (A3), when s>u, the evolution time is one of $t_{1}$ and $\pi -t_{1}$, and one of $t_{2}$ and $\pi -t_{2}$. When the values of *s* and *u* are exchanged, the parameters *a* and *c* remain unchanged, while *b* changes to −b, take the other set of time values. Under the condition $a^{2}+b^{2}-c^{2}\geq 0$, we obtain

(A10) $$\begin{array}{cc} \; & a^{2}+b^{2}+ac=\frac{1}{2}a^{2}+b^{2}-\frac{1}{2}c^{2}+\frac{1}{2}{\left(a+c\right)}^{2}\geq 0,\\ \; & a^{2}+b^{2}-ac=\frac{1}{2}a^{2}+b^{2}-\frac{1}{2}c^{2}+\frac{1}{2}{\left(a-c\right)}^{2}\geq 0,\\ \; & {\left(a^{2}+b^{2}+ac\right)}^{2}-{\left(\sqrt{b^{2}\left(a^{2}+b^{2}-c^{2}\right)}\right)}^{2}=\left(a^{2}+b^{2}\right){\left(a+c\right)}^{2}\geq 0,\\ \; & {\left(a^{2}+b^{2}-ac\right)}^{2}-{\left(\sqrt{b^{2}\left(a^{2}+b^{2}-c^{2}\right)}\right)}^{2}=\left(a^{2}+b^{2}\right){\left(a-c\right)}^{2}\geq 0, \end{array}$$

which indicates that Equation (A6) is the sole condition for $t_{1}$ and $t_{2}$ to be non-trivial solutions.

## Appendix B. Proof of the Comparison of Evolution Times Between the Two Systems

We fix $\sqrt{su}=k$ and ω=Δ, so that sinα=sinβ. From Equations (9) and (21), we have

(A11) $$\begin{array}{cc} \; & \cos^{2}\alpha \langle \psi_{1}|U_{PPH}^{†}U_{PPH}|\psi_{2}\rangle \\ = & \sin^{2}\left(\omega t\right)\left(-\frac{s+u}{\sqrt{su}}\sin\alpha +\frac{s^{2}+u^{2}}{2su}\cos\varepsilon \right)+\frac{u-s}{2\sqrt{su}}\cos\alpha \sin\left(2\omega t\right)\\ + & \left(2\sin^{2}\alpha -1\right)\sin^{2}\left(\omega t\right)\cos\varepsilon +\cos^{2}\alpha \cos\varepsilon , \end{array}$$

and

(A12) $$\begin{array}{cc} \; & \cos^{2}\beta \langle \psi_{1}|U_{PT}^{†}U_{PT}|\psi_{2}\rangle \\ = & 2\sin^{2}\left(\Delta t\right)\sin^{2}\beta \cos\varepsilon -2\sin^{2}\left(\Delta t\right)\sin\beta +\cos^{2}\beta \cos\varepsilon . \end{array}$$

We define the function f(x) with $x=\sqrt{\frac{u}{s}}>0$

(A13) $$\begin{array}{cc} f(x) & =-\left(\frac{1}{x}+x\right)\sin^{2}\left(\omega t\right)\sin\alpha -\left(\frac{1}{x}-x\right)\frac{\sin(2\omega t)\cos\alpha }{2}\\ \; & +\left(x^{2}+\frac{1}{x^{2}}\right)\frac{\sin^{2}\left(\omega t\right)\cos\varepsilon }{2}\\ \; & =A\left(\frac{1}{x}+x\right)+B\left(\frac{1}{x}-x\right)+C\left(x^{2}+\frac{1}{x^{2}}\right), \end{array}$$

where

(A14) $$\begin{array}{cc} \; & 0<\omega t<\pi /2,\\ \; & A=-\sin^{2}\left(\omega t\right)\sin\alpha <0,\\ \; & B=-\frac{\sin(2\omega t)\cos\alpha }{2}<0,\\ \; & C=0<\frac{\sin^{2}\left(\omega t\right)\cos\varepsilon }{2}<1. \end{array}$$

The expression f(x)−f(1) denotes the difference between $\langle \psi_{1}|U_{PPH}^{†}U_{PPH}|\psi_{2}\rangle$ and $\langle \psi_{1}|U_{PT}^{†}U_{PT}|\psi_{2}\rangle$. We fix the evolution time *t* and prove that for x<1

(A15) f(x)−f(1)<0,

and for x>1

(A16) f(x)−f(1)>0.

Next, we use Taylor series expansion of f(x) at x=1 is given by

(A17) $$\begin{array}{cc} \; & \frac{1}{x}=2-x+o\left(\delta \right),\\ \; & \frac{1}{x^{2}}=3-2x+o\left(\delta \right), \end{array}$$

where δ=|x−1|, we obtain

(A18) $$\begin{array}{cc} f(x)-f(1) & =o\left(\delta \right)A+\left(2-2x+o\left(\delta \right)\right)B+\left(x^{2}-1\right)C+\left(2-2x+o\left(\delta \right)\right)D\\ \; & =o\left(\delta \right)\left(A+B+C\right)+2\left(1-x\right)B+{\left(x-1\right)}^{2}C. \end{array}$$

When δ is sufficiently small, we have f(x)−f(1)>0 for x>1, whereas f(x)−f(1)<0 for 0<x<1.

## Appendix C. Further Numerical Simulations

In this appendix, we present additional numerical simulations to supplement the main text. In Figure A1, Figure A2 and Figure A3, we set ε=π/4,π/7,π/10, respectively. The results show that the states $U_{PPH}|\psi_{1}\rangle$ and $U_{PPH}|\psi_{2}\rangle$ become orthogonal faster than $U_{PT}|\psi_{1}\rangle$ and $U_{PT}|\psi_{2}\rangle$.

In Figure A4, Figure A5 and Figure A6, we also choose ε=π/4,π/7,π/10, respectively. We find that when s>u, $U_{PPH}|\psi_{1}\rangle$ and $U_{PPH}|\psi_{2}\rangle$ become orthogonal faster than $U_{PT}|\psi_{1}\rangle$ and $U_{PT}|\psi_{2}\rangle$. Conversely, when s<u, $U_{PT}|\psi_{1}\rangle$ and $U_{PT}|\psi_{2}\rangle$ become orthogonal faster than $U_{PPH}|\psi_{1}\rangle$ and $U_{PPH}|\psi_{2}\rangle$.
